# Supplementary material for: Can the detection dog alert on COVID-19 positive persons by sniffing axillary sweat samples? A proof-of-concept study
Source: PLoS One. 2020 Dec 10;15(12):e0243122. doi: 10.1371/journal.pone.0243122 (PMC7728218; doi:10.1371/journal.pone.0243122)
Supplement: Attachment — Submitted filename: Response to Reviewers (Revision #2) v2.docx [file pone.0243122.s003.docx]

PONE-D-20-17256R1
Can the detection dog alert on COVID-19 positive persons by sniffing axillary sweat samples?
A proof-of concept study
PLOS ONE

Dear Dr. Grandjean,

Thank you for submitting your manuscript to PLOS ONE. After careful consideration, we feel that it has merit but does not fully meet PLOS ONE’s publication criteria as it currently stands. Therefore, we invite you to submit a revised version of the manuscript that addresses the points raised during the review process.
Two reviewers are still requiring some clarification or some revision of your conclusions and importantly recognize some indications of the limits of your study.

Please submit your revised manuscript by Nov 13 2020 11:59PM. If you will need more time than this to complete your revisions, please reply to this message or contact the journal office at plosone@plos.org. When you're ready to submit your revision, log on to https://www.editorialmanager.com/pone/ and select the 'Submissions Needing Revision' folder to locate your manuscript file.

Please include the following items when submitting your revised manuscript:

- A rebuttal letter that responds to each point raised by the academic editor and reviewer(s). You should upload this letter as a separate file labeled 'Response to Reviewers'.
- A marked-up copy of your manuscript that highlights changes made to the original version. You should upload this as a separate file labeled 'Revised Manuscript with Track Changes'.
- An unmarked version of your revised paper without tracked changes. You should upload this as a separate file labeled 'Manuscript'.

If you would like to make changes to your financial disclosure, please include your updated statement in your cover letter. Guidelines for resubmitting your figure files are available below the reviewer comments at the end of this letter.

If applicable, we recommend that you deposit your laboratory protocols in protocols.io to enhance the reproducibility of your results. Protocols.io assigns your protocol its own identifier (DOI) so that it can be cited independently in the future. For instructions see: <http://journals.plos.org/plosone/s/submission-guidelines#loc-laboratory-protocols>

We look forward to receiving your revised manuscript.

Kind regards,

Nadine Ravel
Academic Editor
PLOS ONE


[Note: HTML markup is below. Please do not edit.]

Reviewers' comments:

Reviewer's Responses to Questions

**Comments to the Author**

1. If the authors have adequately addressed your comments raised in a previous round of review and you feel that this manuscript is now acceptable for publication, you may indicate that here to bypass the “Comments to the Author” section, enter your conflict of interest statement in the “Confidential to Editor” section, and submit your "Accept" recommendation.

Reviewer #1: (No Response)

Reviewer #2: All comments have been addressed

Reviewer #3: (No Response)

2. Is the manuscript technically sound, and do the data support the conclusions?

The manuscript must describe a technically sound piece of scientific research with data that supports the conclusions. Experiments must have been conducted rigorously, with appropriate controls, replication, and sample sizes. The conclusions must be drawn appropriately based on the data presented.

Reviewer #1: Partly

Reviewer #2: Yes

Reviewer #3: Partly

3. Has the statistical analysis been performed appropriately and rigorously?

Reviewer #1: I Don't Know

Reviewer #2: Yes

Reviewer #3: No

4. Have the authors made all data underlying the findings in their manuscript fully available?

The [PLOS Data policy](http://www.plosone.org/static/policies.action#sharing) requires authors to make all data underlying the findings described in their manuscript fully available without restriction, with rare exception (please refer to the Data Availability Statement in the manuscript PDF file). The data should be provided as part of the manuscript or its supporting information, or deposited to a public repository. For example, in addition to summary statistics, the data points behind means, medians and variance measures should be available. If there are restrictions on publicly sharing data—e.g. participant privacy or use of data from a third party—those must be specified.

Reviewer #1: No

Reviewer #2: Yes

Reviewer #3: Yes

5. Is the manuscript presented in an intelligible fashion and written in standard English?

PLOS ONE does not copyedit accepted manuscripts, so the language in submitted articles must be clear, correct, and unambiguous. Any typographical or grammatical errors should be corrected at revision, so please note any specific errors here.

Reviewer #1: No

Reviewer #2: Yes

Reviewer #3: Yes

6. Review Comments to the Author

Please use the space provided to explain your answers to the questions above. You may also include additional comments for the author, including concerns about dual publication, research ethics, or publication ethics. (Please upload your review as an attachment if it exceeds 20,000 characters)

Reviewer #1: The authors have considerably improved the manuscript, by providing significant information that was lacking in the previous version, and by improving the structure of the manuscript. For this kind of study, and especially due to the potential impact its results may have on the current management of the Covid-19 disease, it remains essential that the methodology is carefully discussed, so that the validity of the conclusions can be properly evaluated.

We thank the reviewer for her/his comments.

Of note, the Introduction section has been re-ordered.

In the current version, the additional details provided have elicited further questions about the validity of the conclusions:

1) The pairing between COVID-19 positive and COVID-19 negative odor donors represents a strong limitation in this study. Whereas the Lebanese groups are comparable, age differences between both groups in Paris is considerable: as body odor differs with age (to human noses as well as in terms of chemical composition: Mitro et al 2012 “The smell of age: perception and discrimination of body odors of different ages”, Chen & Haviland-Jones 2000 “Human olfactory communication of emotion”, Haze et al 2001 “2-Nonenal newly found in human body odor tends to increase with aging”), this may interfere with the dogs’ discrimination. The results suggest that it may introduce noise that alters the dog’s performance, since French dogs had lower success rates than Lebanese dogs. In future studies, targets and controls should at least be paired for sex and age.

We thank the reviewer and we added this comment in the Discussion section (9^th^ paragraph).

Another even more important limit is the fact that COVID-19 negative donors were partly people hospitalized for another reason than COVID-19. Meaning that they probably are on medication, and on medication that differs from the one taken by COVID-19 positive patients. How it may impair dogs’ performance or bias there responses towards the COVID-19 positive sample is currently difficult to predict in the absence of further information regarding patients’ treatment.

We added this comment in the Discussion section (10^th^ paragraph).

2) Mocks. The authors now explain that there were 3 or 4 cones in the lineups, including one COVID-19 positive sample and zero, one or two mocks. First, I assume this was zero or one for the 3-cones lineups, and zero to two mocks for the 4-cones lineups, i.e. there was always at least one COVID-19 negative sample in the lineup. Please confirm and write it explicitly in the text. Second, what was the rationale for using mocks? This is potentially a problem, since the test is much easier for the dog when they have 2 mocks plus 2 body odors (i.e., one positive and only one negative sample): they actually are discriminating between 2 and not 4 samples (the level of successfully answering by chance is much higher), since they were trained before to discriminate sweat samples from mocks (step 3 of the training).

First, the mocks were present in the lineups of three dogs out of the six dogs. Second, there was always at least one COVID-19 negative sample in the lineup. We clarified these points in the “Testing protocol” of the manuscript.

The presence or absence of mocks in the lineup depended on the way the dog usually work with its handler for explosive detection, search and rescue, or for colon cancer detection. Gun, Oslo, Bella and Jacky are used to work without any mock whereas Guess and Maika were used to work with mocks. Because the dogs were active working dogs before this study, we did not want to interfere with the way they usually work. Three trials out the 47 trials of Gun included one mock: these trials were the last ones out of the 47 trials of Gun, and one mock was included in the lineup because of the lack of COVID-19 negative samples. We decided to include these three trials for Gun in the Results section (in which the success rate was 100%) because we wanted to provide all the data in the new Table 2 which were already present in Table 2 of the previous version of the manuscript. However, it is ok for us if the Editor or the reviewer wants to remove the results calculated from the 3 trials of Gun.

We also modified the presentation of the results in the Result section, by describing precisely the number of mocks in the lineups for each of the six dogs.

We agree that the test would be easier for the dog if there were 2 mocks out of 4 cones, compared to 1 or 0 mock out of the 4 cones. Because of this issue, we modified the calculation of the random choice proportion by removing the number of mocks from the calculation (ie, if a 4-cone lineup contained one COVID-19 positive sample, one COVID-19 negative sample, and two mocks, the random choice proportion is now 50%). We strongly modified the presentation of our results in Table 2, as well the text in the Results section. We also added a paragraph in the Discussion section regarding this issue raised by the reviewer. Most of the times (except one time), the lower bound of the 95% confidence interval of the estimated success rates remained higher or equal to the random choice proportion. The lower bound of the 95% confidence interval of the estimated success rate of 100% however included the value of random choice proportion because this success rate was calculated from 3 trials only, for Gun. The message of our manuscript remains the same after removing the mocks in the new calculation of the random choice proportion, as it provides some evidence that “detection dogs may be able to discriminate between sweat samples from symptomatic COVID-19 individuals and those from asymptomatic COVID-19 negative individuals” (as now stated in the Abstract).

We do hope that we have answered the point raised by the reviewer.

3) Where was the dogs’ testing performed? Why did one dog have only 3 cones, and not 4 as the others?

The dogs’ testing was performed in the specific and dedicated room in which cones were positioned.

Gun worked with three cones instead of four like the other dog because of its personality: when Gun started to work with four cones, it presented an instable behavior and too much excitation. The Gun’s handler decided to finally work with 3 cones instead of four. Because the random choice proportion was calculated based on the number of cones for each dog (and now, the number of COVID-19 negative and positive samples in the lineup – see previous comment), we do think that the results were not biased for Gun (because Gun worked on 3-cone lineups instead of on 4-cone lineups).

4) Repetition of sample presentations during testing may also be an issue, as raised by the other reviewers. The authors response to this limit was not convincing: rather, they could test whether the performances increased on the second or third repetition compared to the first presentation. In fact, the results section is very short: the authors could very well test some of the possible bias arising from methodological flaws and present them here. For example, in addition to testing the effect of sample repetition, I suggest that they i) test whether lineups including only COVID-19 positive and healthy COVID-19 negative (no sick COVID-19 negative) elicit higher/lower performance, ot ii) test whether one sampling method provided better performances than the other. On the latter point, the fact that “two sampling supports were used because at the time to the beginning of the proof-of-concept study, we did not know which one of the two sampling supports was the most effective” should appear in the manuscript, otherwise no one will understand why you did so. This study being a proof-of-concept study, this is fine to mention that. And it would be nice to have some info regarding effectiveness of both methods.

As requested by the reviewer, we calculated the success rates according to the rank of the presentation of the COVID-19 positive sample to the dog, for each dog from Paris site (we do not have the data of the Lebanese site allowing us to perform the same statistical analyses, because of the recent exploding event in Beirut). The results are also presented in a new Table we inserted into the manuscript (Table 3):

|  | First, second, or third presentation of the COVID-19 positive sample | Number of trials | Correct identifications | Success rate [95% CI] |
| --- | --- | --- | --- | --- |
| Guess | 1^st^ | 24 | 22 | 92% [73%-99%] |
|  | 2^nd^ | 24 | 18 | 75% [53%-90%] |
|  | 3^rd^ | 4 | 3 | 75% [19%-99%] |
| Maika | 1^st^ | 23 | 20 | 87% [66%-97%] |
|  | 2^nd^ | 26 | 20 | 77% [56%-91%] |
|  | 3^rd^ | 6 | 5 | 83% [36%-100%] |
| Gun | 1^st^ | 24 | 20 | 83% [63%-95%] |
|  | 2^nd^ | 23 | 22 | 96% [78%-100%] |
| Oslo | 1^st^ | 21 | 19 | 90% [70%-99%] |
|  | 2^nd^ | 10 | 10 | 100% [69%-100%] |
| CI: Confidence interval | | | | |

As the reviewer will see for the four Parisian dogs, the success rate seems not related to the replication number of the COVID-19 positive sample: for Guess and Maika, the success rate is even higher when the COVID-19 positive sample was presented to the dog for the first time compared to the second and third times. For the two other dogs, even if the success rate was lower when the COVID-19 positive samples were presented for the first time to dogs compared to for the second time, the success rates were higher than 80%, and their lower bound of the 95% confidence interval were still above the maximum value of the random choice proportion in our study (50%). These results support the hypothesis according to which the olfactory memory of dog did not play a major role, if any, in the discrimination task in our study.

We thank the reviewer for having raised this point, since these new results seem to weaken the limitation due to the replication of the COVID-19 positive samples. We added in the Results section these results. And we modified the Discussion section, 7^th^ paragraph.

We do not have the information according to which COVID-19 negative individuals are sick or healthy. Most of them are patients in the hospital where the COVID-19 positive sample was collected, so they might be “sick”. Therefore, had we had the information, we would not have enough statistical power by using healthy COVID-19 negative individuals only.

As recommended by the reviewer, we added in the 3^rd^ paragraph of the “Samples” part the following sentence: “Two types of sampling supports were used because at the time to the beginning of this proof-of-concept study, it was not known which one of the two sampling supports was the most effective.” Actually, we do not want to present the comparison between the two types of support for two reasons: (1) it was not the objective of the study and the study was not designed to do so (and interpreting an association after it is statistically tested could be seen as HARKing (Kerr NL. HARKing: hypothesizing after the results are known. Pers Soc Psychol Rev 1998), and (2) we plan to set up a study of which one of the objective is actually to compare the two types of supports.

5) Last paragraph of introduction: you mention the recommendations on how to proceed to rigorously test your research question but we expect to read how you study is positioned within these recommendations. This is currently not the case.

It would be uneasy to read how the study is positioned right after the paragraph which mentioned the recommendations at the end of the Introduction section. Furthermore, by reading the Material and Methods section, the reader is able to know how the study is positioned. Finally, in the Discussion section, we already mentioned each of the criteria listed at the end of the Introduction section:

Replication of the study samples: 7^th^ paragraph

Comparability criteria: 8^th^ → 11^th^ paragraph

Single/double blindness: 13^th^ paragraph

Randomisation: 13^th^ paragraph

We however modified the paragraphs in the Discussion section regarding the replication of the study samples and regarding the comparability criteria, based on the previous comment by the reviewer.

6) There are erroneous references to the bibliography. First, page 4 “sweat does not appear to be a route of excretion for SARS-CoV-2 virus [44, 45]”. In these papers, sweat is not mentioned. Instead, it appears that in the absence of scientific study on that topic, we don’t know whether sweat is able to convey the virus or not, see the following paper: Propper, R.E., 2020. Is sweat a possible route of transmission of SARS-CoV-2? Second, page 6: “SARS-CoV-2 virus does not survive more than a few hours on cotton and disposable gauze [52]. A more recent study concludes that absorbent materials like cotton and gauze are safer than no-absorptive materials for protection from SARS-CoV-2 infection [53].” References 52 and 53 are about SARS-CoV and not SARS-CoV-2, and about the comparison between virus survival on cotton gown versus disposable (polypropylene) gown. Reformulation is needed.

We thank the reviewer for these remarks. We modified the concerned paragraphs in the Material and Methods section (2^nd^ paragraph in the “Safety of the dogs regarding SARS-CoV-2 virus infection” part). However, one sentence of the abstract of Ren et al. states: “Absorbent materials like cotton are safer than unabsorbent materials for protection from virus infection”. “Polypropylene” term was used in the Lay et al. paper, but when we cited the Lay’s paper in our manuscript, we did not refer to the comparison between cotton gown and disposable gown. We therefore do not see the rationale to mention in our sentences “disposable (polypropylene) gown”.

Overall, to make this study publishable, I would recommend to formulate the discussion and abstract even more cautiously. The conclusions are still too affirmative according to the numerous limitations. It should be explicitly stated that more research is need in the future, addressing a number of limitations present in this study – and then all limitations should be carefully listed. Also, the manuscript has clearly not been proofread by an English native speaker, as many language and wording imperfections remain.

The end of the abstract is now “However, due to the limitations of this proof-of-concept study (including using some COVID-19 samples more than once and potential confounding biases), these results must be confirmed in validation studies”.

We also modified the Discussion section by pointing out even more the limitations of our study. We do not know now how to list more carefully the limitations of our study. We do hope that we have answered the issues listed above by the reviewer.

The manuscript has been corrected by a English native speaker.

Reviewer #2: Review of the Revision R1

The Authors have thoroughly addressed my remarks and comments, both in their responses to my review and in the revision. The plenty of changes to the manuscript makes the tracking of them hardly possible, thus the revision could be considered almost as a new manuscript. Obviously the revision substantially benefits from such considerable changes.

The Authors are aware that in order to avoid confounding factors, control samples must be in all aspects comparable to positive samples, except for the disease status. This was now evident from the lines 131-132, lines 142-145 and 350-365 of the revision.

We thank the reviewer for her/his comments.

Of note, the Introduction section has been re-ordered.

In lines 371-373 of the revision the Authors properly address the problem of differences in the amount of sweat collected from COVID positive and negative donors, due to possible fever, which is a typical COVID-19 symptom. This issue was my main concern. In the revision the authors inform that the amount of sweat was not recorded and it can not be excluded that it could be a confounding factor. This statement is correct, however, further discussion in lines 374-378 including citation of Concha et al. (2019), is not quite pertinent to the problem I would like to point out. In the study of Concha et al. (2019) on detection threshold of amyl acetate, the dogs had to discriminate between amyl acetate diluted in mineral oil at different concentrations and pure mineral oil as control. There were defined qualitative chemical differences between target odour and control odour up to a dilution of amyl acetate which was for dogs not distinguishable from mineral oil. On the contrary, in the present study it is still not known which volatile organic compounds in the sweat of COVID-positive patients make that the dogs are able to discriminate the sweat odour of COVID positive from negative humans. Also, the saturation of olfactory receptors by a great amout of the odorant (revision lines 377-378), is not pertinent to the problem of the possible differences in the amount of sweat in the samples. I wanted only to point out that potential differences in the amount of sweat could be a confounding factor and/or could be a cue for dogs. I suggest to delete the passage in revision lines 374-378.
An important information was given only in the response to my review, namely that the samples contained approximately 75 mg sweat and no differences were found between positive and negative samples e.g. due to more sweating in fever. In order to avoid doubts of the readers, this information should be given not only in responses to the review, but first of all in the revision, in the paragraph dedicated to sample collection.

We modified the last part of the concerned paragraph in the Discussion section (11^th^ paragraph), as well as the Material and Methods section accordingly (3^rd^ paragraph in the “Samples” part).

In the revision the Authors have resigned from the 4 Ajaccio dogs and 21 sweat samples collected in Ajaccio. This was convincingly justified in Authors’ response to the Reviewers #1 and #3. As to the number of dogs used, the Authors in their explanations to the Reviewers #3 inform that now they used six dogs (for the real tests) and they did not mention anymore the number of 14 dogs used for the training. The Authors now explain that „…it added a non relevant information…”. However, in my opinion it is just an important information, because it shows that not all dogs are able to meet the training or qualification criteria for this task. An information on how many dogs out of all candidates that started the training, were qualified for the test, would be useful for other researchers or dog trainers who will intend to continue or to repeat such experiments.

We thank the reviewer for this comment. Initially, there was 14 dogs which participated to the training sessions. Out of these dogs, six of them were finally considered as ready by their handler. The reason why 8 dogs did not participate to testing session is because the training started too late regarding the date of the beginning of the testing session which could not have been postponed (we needed to start the testing session as soon as possible because of the urgency the COVID-19 detection dogs subject). We consequently modified the Material and Methods section (“Canine resources”, “Training protocol”, “Testing protocol”), the Results section, as well as the Discussion section (14^th^ paragraph). In the Results section, we now explain why eight of the 14 dogs did not participate to the testing session, and we now present the six participating dogs in the Results section (instead of in the Material and Methods section, as it was initially). We modified the 14^h^ paragraph of the Discussion section, by presenting the lack of time to train the dogs as the first reason why the length of training among our 6 dogs which finally participated to the testing session is shorter than previously reported ones. We also added the following information which could be useful for other researchers or dog trainers who will intend to continue or to repeat such experiments: “In operational settings, it is recommended to expect around six weeks to train a COVID-19 detection dog”.

As to the qualification criterion, the Authors’ information that „…The dog was considered as trained and ready for the testing session by the subjective opinion of the handler, based on the behavior of the dog….” – is acceptable, though for the sake of scientific accuracy in future experiments more precise quantitative criteria would be recommended.

We agree with the reviewer that in order to improve the Quality of Scientific Research, the whole protocol should be written (Barba, Science, 2016). However, training a dog is a full procedure by itself involving a strong interaction between the dog and its handler (Hoummady, Appl Anim Behav Sci, 2016). Therefore, judging that the dog is ready to work necessarily requires a subjective judgment by its handler. Elliker et al. wrote in their 2014 paper “When the dog was able to identify the scent hole containing the CaP more frequently than expected by chance it was moved to the formal test stage”, but it was not a recommendation but rather the way that the authors considered their dogs as ready. In the two other papers in which recommendations are written (Edwards, 2017 and Johnen 2017), we did not read recommendations in order to objectively judge that a dog is ready to work. Finally, Edwards et al. wrote in their 2017 paper: “As others have pointed out, there are no standards for training disease detection animals (e.g., Jezierski et al., 2015; Oh et al., 2015; Walczak et al., 2012), and the practice of sharing procedures may be instrumental in the development of such standards.”

It is not clear to me why the Authors think the calculation of the sensitivity and specificity was not possible ? (revision lines 276-277).

It depends on how the outcome of a trial in the lineup is assessed. The authors apparently considered their lineup as a simultaneous lineup, where the dog makes a choice out of 3 or 4 samples. This approach seems to be more „convincing” because the theoretical probability of the correct indication by chance is relatively low (1/4 or 1/3). However, the actual probability of correct choices by chance depends on how many samples the dog had really sniffed. The not sniffed samples do not count for calculation of correct hits by chance. Therefore the Authors are not correct, claiming in their explanations that this probability is still 25% if the dog hits correctly on the first cone, without sniffing the remaining cones. Thus, the probabilities given in the last right-hand column of the Table 2 are not constant but variable from trial to trial.

We do not agree with the reviewer.

Here is an example to justify the reason why we do not agree.

Below is a play of four cards.

If we ask a 7-year old child to pick the queen of diamonds, by telling him that this queen of diamonds *is* in the play of cards, and that he has only one trial, what will the child do?

If the child has played to cards for a long time, he might start to look at the first card from the left without looking at the other, and pick up the first card because he recognizes it right away.

If the child played only one or two times to cards before, he might start to look at the first card from the left then the second, then the third, then the fourth, and finally pick-up the first because he knows that the color is read, and he knows the shape of a heart.

If the child never played to cards before, he might start to look at all the cards and pick-up one card by chance.

Our point is that, regardless of the way the child is going to look at the cards (only one card, or two, three or four), the probability that the child chooses the queen of diamond by chance only is 1/4, because he knows that the queen of cards is in the play of cards, and that he will have only one trial.

Actually, if one asks to an adult to pick the queen of cards, his eyes will right away (in less than one millisecond) look at all the cards and pick the right one.

The analogy is that child’s eyes is the dog’s nose, and the fact that the child knew that there was the queen of the diamonds in the play of cards is the fact that the dog knows that there is only one COVID-positive sample in the lineup.

However, to answer the comment #2 of the reviewer #1, we modified the calculation of the random choice proportion by removing the mocks in the lineup from the calculation.

The other approach is a sequential lineup where the dogs are considered to make separate and independent yes/no decisions to each sniffed sample, at the constant 50% probability of correct hits by chance. The sequential lineup is believed to be statistically more sound. If the Authors would consider their lineup as the sequential one, all sniffed but not indicated cones with negative samples are „true negative”, all correct hits on positive samples can be regarded as „true positive”, and the indications labelled in Authors’ protocols as “failures” - are „false positive”. Since in the present protocol „…the dog was previously trained to detect one and only one COVID-19 positive cone, the dog was left free to sniff the cones before marking one cone….” and „….Once a cone was correctly or incorrectly marked, the trial was stopped..(revision lines 258-260) – it means that in the protocol like this, there were no „false negatives” (f.n.=0). Assuming that f.n. =0, the calculated sensitivity would be 100% [Ʃ true positive / Ʃ (true positive + false negative). The specificity for some dogs would be <100% [= Ʃ true negative / Ʃ (false positive + true negative)]. However, from the statement in revision lines 258-260, one can suspect that there was a bias toward reducing „false negative” responses (a miss to indicate the target sample), since the handler knew, and the dogs were trained, that in each trial one of the samples must be the target to be indicated. A problem would be when it comes to testing samples of unknown health status in real scenario. The way to avoid this problem would be either to introduce from time to time a „zero” or „blank” trial (with all negative samples in the lineup), or to set a limit for how many times the dog is allowed to sniff positive samples without indicating, before it eventually hit them. For example if the dogs has sniffed the positive sample 2 or 3 times without indicating, the outcome can be classified as the „false negative”.

Since the aim of the study was only to confirm that dogs are able to discriminate COVID positive and negative sweat samples, the term „Success rate” used by the authors, is acceptable, but for the sake of scientific accurracy in future studies the Authors should be able to calculate the sensitivity and specificity.

We thank the reviewer for her/his comments because it enabled us to better clarify the reason why we were not able to calculate sensitivity and specificity. The lineup is a “simultaneous” lineup, and not a “sequential” lineup. We totally agree with the reviewer that had the lineup been a sequential one, we would have had to provide sensitivities and specificities. In a simultaneous lineup, the sample is not evaluated individually by being marked or not, one by one, since we asked the dog to mark only one cone. In our validation study we are implementing now, we actually use the lineup as a sequential one in order to calculate sensitivities and specificities, as recommended by the reviewer.

We modified the Material and Methods section (end of 1^st^ paragraph of the “testing protocol” part) and the Discussion section (6^th^ paragraph).

It is worth underlining that the revision is better structured than the first submission. The concluding remarks are more cautious and the references are updated.

We thank the reviewer for her/his comments.

Minor remarks

The terms „odour detection” and „odour discrimination” are frequently used interchangeably, however, in the context of this work I suggest to use the wording „odour discrimination” rather than „odour detection”. Odour detection refers rather to searching for unknown location of odour source in a field setting. In the odour lineup like this, the dogs are trained to visit the odour locations (cones) which are visible, and their task is to compare and to discriminate between the target and control odours.

We modified the manuscript accordingly when the “detection” term did not refer to “detection dogs”, or to the literature. However, we did not systematically replace “detect” by “discriminate” because sometimes in the manuscript, we really wanted to talk about “to search for unknown location of odor source in a field setting”, where the field is composed of cones in one room.

In lines 344 and 352 the year of the publication should be completed for more clarity. This is particularly important when two or more papers of the same first author are cited.

We modified the sentence in which “Williams et al.” was written (6^th^ paragraph), and we added “in their paper published in 2012” when we cited the paper by Wolczak et al.

Line 373 „COVID-19 negative” should be inserted for more clarity.

We thank the reviewer and we modified the sentence as suggested.

To sum up, only few amendments (minor revision), as listed above, should be made to the text to be fully acceptable for publication in the PLoS ONE.

We thank the reviewer for her/his comments.

Reviewer #3: Thank you to the authors for taking into account the previous comments from the three reviewers, providing very detailed responses to the points raised. The revised manuscript is much improved, provides a more balanced appraisal of the study and is much clearer to read. Some of the additional information that has been added has raised further questions and comments, detailed below, but I think the manuscript would be appropriate for publication if these can be addressed.

We thank the reviewer for her/his comments.

Of note, the Introduction section has been re-ordered.

General comments

- The general use of English language and grammar has greatly improved in this revision. However, there are still a few typographical errors and language issues in places that need addressing with a thorough proof reading.

The revised manuscript has now been corrected by native English speaker.

- Throughout the manuscript (title, abstract, discussion etc.) suggest you clarify that you were testing a discrimination of *symptomatic* SARS-CoV-2 positive vs asymptomatic SARS-CoV-2 negative. This is an important distinction, as symptomatic individuals may produce confounding odours for a wide variety of reasons.

We added the “symptomatic” and “asymptomatic” terms all the time it seemed necessary (not all the time in order to make the reading too difficult).

Abstract
- Line 49 “The aim … was to evaluate if a sample of sweat produced by COVID-19 50 positive individuals (SARS-CoV-2 positive) can be detected by trained detection dogs among 51 sweat samples produced by COVID-19 negative individuals.” This wording suggests that dogs were evaluated using just a single (one) SARS-CoV-2 PCR positive sample. The line needs clarifying; I think you mean to say something like: “evaluate if dogs could detect individual samples of sweat produced by COVID-19 50 positive individuals (SARS-CoV-2 PCR positive) presented in a line up amongst sweat samples produced by COVID-19 negative individuals”?

We agree that the sentence was not clear enough, and modified it accordingly to the reviewer#3 and also according to one comment of the reviewer #2.

- I think you need to state in the abstract that there are limitations to the study (e.g. processes for collecting positive and negative samples were not completely identical, the trial stage was not fully double blind, some samples were re-used several times during the trial stage) and that a more rigorous validation study is ideally needed to confirm the results.

We modified the abstract accordingly. However, we did not mention the limitation of the lack of double blindness of the study because (1) the fact the data recorder was the only one person who knew the location of the COVID-19 positive sample without any eye contact between the dog, while staying behind his table on the back of the room is not a strong limitation (compared to the replication of some positive samples and confounding biases). Furthermore, in the abstract, we would have used too many words to explain the difference between the full double blindness and what occurred in our study. Instead, we pointed out the strength of our study by mentioning that the dog and its handler were blinded to the location of the positive sample. We however modified the 13^th^ paragraph of the Discussion section: “It should however be noted that the study should ideally be double-blind (i.e., both the handler and the data recorder are blinded to the sample positions), while being able to reward the dog using various methods (Elliker, 2014)”.

Introduction
- For balance, I think you should mention that not all studies testing canine detection of disease have shown positive results. There is currently very little evidence relating to canine detection of virus odours, particularly using live subjects (you cite one example using cell cultures).

We modified the Introduction section in order to balance the positive results provided by the published studies cited in the manuscript: “As pointed out by Edwards et al. in 2017, “one of the largest threats to the validity of the results from [olfactory detection dog] studies is the possibility that systematic differences, other than disease status, between positive and control samples in both training and testing phases were responsible for the obtained results.” (Edwards, 2017). Therefore, the current amount of evidence suggesting that dogs can be used as a complementary tool to detect infectious and non-infectious diseases may not be as high as the number of published studies on the subject suggests”.

We do hope that we have answered the point raised by the reviewer.

Materials and Methods

Samples
- Line 157. I think you need to briefly discuss in your discussion section/conclusions the possible limitation that SARS-CoV-2 positive samples were collected using full PPE whereas negatives were not. It could have produced a consistent difference in odour that confounded the results. Were the same brands of gloves used for collecting positive and negative samples? If different brands/types were consistently used for positives and negatives, it’s a major limitation as the dogs may have trained on different glove odours.

Full PPE was not systematically used for COVID-19 negative individuals. However, there was no contact between the equipment and the samples: the gloves were the only objects in contact with the samples. And the brands of gloves used for COVID-19 positive and COVID-19 negative individuals from the same hospital were the same. We added this point (the brands of the gloves) in the Material and Method section (“Sample” part). The comparability criteria was therefore fully met for the brands of the gloves between COVID-19 positive and negative individuals. We therefore did not want to add this point in the Discussion section, since it does not add another more limitation of our study.

- Line 177. Were the two coolers identical (same brand, from the same source etc.?). Worth stating if this was the case. If not, it’s a limitation as dogs may have trained on the odour of two different coolers.

Yes, the coolers were of the same brand. This information was added in the manuscript (« Sample » part in the Material and Methods section).

Safety of the dogs regarding SARS-CoV-2 virus infection

- Line 216. Should read “non-absorptive”

Based on a comment by a previous reviewer, we modified this sentence and we used the “unabsorbent” word, as in the Ren et al. 2020 paper (“Absorbent materials like cotton are safer than unabsorbent materials for protection from virus infection”).

Testing protocol

- Line 248. Please clarify the minimum number of SARS-CoV-2 negative samples that were placed out each time. If zero negatives samples were placed out, that would not be valid discrimination data and should be excluded from the analysis/results. If 1, 2 or 3 samples were placed out, please clarify how this was accounted for in the statistical analysis.

As we answered the same question to reviewer #1, we now take into account the number of mocks in the lineups (see our response to reviewer #1). Briefly, there was always at least one COVID-19 negative sample in the lineup. But because mocks and COVID-19 negative samples may not be considered equally by the dogs, we now present the results (the estimated success rates and the random choice proportion) according to the number of mocks in the lineups (new Table 2). Of note, for 3 dogs out of 6, there was no mock in the lineup. The number of mocks in the lineup is now removed from the calculation of the random choice proportion. The results remained unchanged (ie, the lower bound of the 95% confidence interval of the new success rates were higher or equal to the random choice proportion), except for 3 trials of one dog (Gun) containing 1 mock in the lineups: the success rate was 100%, but the very low sample size (n=3) dramatically increased the width of the 95% confidence interval. We modified the manuscript accordingly (in the Material and Method, Results, and Discussion sections).

Statistical analysis

- Line 275. As per the abstract, this wording suggests that dogs were evaluated using just a single (one) SARS-CoV-2 positive sample. The line needs clarifying.

We modified the sentence as requested.

- As mentioned in the testing protocol comment, some additional detail is needed on how the statistical analysis accounted for the different number of negative samples placed out. If some line-ups included zero negatives, these trials should be excluded from the analysis. It is vital that the analysis is based on the number of negatives placed out, not the number of cones. Clearly, dogs would have a greater chance of detecting the positive samples with lower numbers of negatives present. If not all the cones contained negatives the statistical analysis needs to be revised to account for this.

As mentioned earlier, we now take into account the issue raised by the reviewer.

Results
- Line 292. The ages are the wrong way around?

The reviewer was right. We mistakenly switched the mean between the two groups. We thank the reviewer for her/his comments.

Table 2, as stated above, the random choice proportion should be based on the number of negatives placed out, not the number of cones (if not all cones (expected the positive) contained SARS-CoV-2 negative samples).

This is now how we calculated the random choice proportion.

Discussion
- Line 335. This line needs revising to reflect that only positive and negative samples (not “mocks”) should have been included in the statistical analysis.

Since we added one paragraph specifically on this issue right above the one where the reviewer asks for modification, and since in practice, some lineups actually did include mocks (although they are not taken into account anymore in the statistical analyses), we prefer to leave this line as it is, without modification.

- line 389 to 396. Recommend you clarify that the lack of double blinding remains a limitation that should ideally be addressed in a future validation study. Previous studies have used various techniques to reward dogs whilst remaining double blind (e.g. use a live mobile phone conversation with an independent referee who has a list of sample “identities” for each line up).

We modified the end of the paragraph accordingly.

The ideal validation study would be to receive samples from an independent source (e.g. from another organisation or even country) which have not been processed by your research team before being presented to the dogs.

- 411 to 416. Recommend key improvements for the validation trial should include double-blinding and samples prepared and delivered by an independent organisation/source.

We modified the end of the Discussion accordingly.

- Line 412. Should read ‘sensitivity’ not ‘sensibility’.

We corrected this misspelling.
